# Correlation Between the Mineral Contents of Drinking Water and Dental Caries in Mangalore: A Cross‐Sectional Study

**DOI:** 10.1155/ijod/6407047

**Published:** 2026-09-28

**Authors:** Thara Chandran, Audrey D’Cruz, Radhakrishnan Kinattinkara, Mackwin D’Mello, Pushparaja Shetty, Neevan D’Souza, Vishnu Unnikrishnan, Herman Sunil Dsouza

**Affiliations:** ^1^ Department of Public Health Dentistry, AB Shetty Memorial Institute of Dental Sciences (ABSMIDS), Nitte (Deemed to be University), Mangalore, India, nitte.edu.in; ^2^ Pedagogy Park, Vanavidyalayam, Society for Environmental Education in Kerala (Rgd.), Kottemchery, Kerala, India; ^3^ Department of Public Health, Nitte Institute of Allied Health Sciences (NIAHS), K S Hegde Medical Academy, Nitte (Deemed to be University), Mangalore, India, nitte.edu.in; ^4^ Department of Oral Pathology and Microbiology, AB Shetty Memorial Institute of Dental Sciences (ABSMIDS), Nitte (Deemed to be University), Mangalore, India, nitte.edu.in; ^5^ Department of Biostatistics, Nitte Institute of Allied Health Sciences (NIAHS), K S Hegde Medical Academy, Nitte (Deemed to be University), Mangalore, India, nitte.edu.in; ^6^ Manipal Institute of Technology, Manipal Academy of Higher Education, Manipal, India, manipal.edu; ^7^ Department of Radiation Biology and Toxicology, Manipal School of Life Sciences, Manipal Academy of Higher Education, Manipal, India, manipal.edu

**Keywords:** dental caries, DMFT, drinking water, magnesium, minerals

## Abstract

**Background and Objectives:**

Physicochemical properties of dental hard tissues are strongly influenced by the ionic concentration of saliva, which is partly determined by the ionic concentration of drinking water. While the role of fluoride ions in dental caries prevention has been documented, the effects of various other minerals have not been studied. This cross‐sectional ecological study aimed to evaluate the relation between various trace elements in drinking water and dental caries experience among residents of Mangalore Taluk.

**Methodology:**

A multistage sampling method was used to select 31 of the 76 gram panchayats. Dental caries was assessed using the decayed, missing, and filled teeth (DMFT) Index. Drinking water samples were collected from groundwater wells in the study area. A total of 31 water samples were collected, and minerals in the groundwater were analyzed using inductively coupled plasma mass spectroscopy (ICP‐MS). The correlation between the mineral concentration in drinking water and dental caries experience was checked using Spearman’s rank correlation test. A *p*‐value less than 0.05 was considered to indicate statistical significance.

**Results:**

The fluoride concentration in the study area was less than the limits recommended by the World Health Organization (WHO). A statistically insignificant weak positive correlation was found between fluoride in drinking water and dental caries. A significant, moderately negative correlation was identified between potassium and dental caries.

**Conclusion:**

This study suggests the potential protective association of potassium in the prevention of dental caries in a low‐fluoride area of Mangalore. These results suggest that the interplay of minerals in drinking water contributes to the anticariogenic effect rather than single ions.

## 1. Introduction

The provision of safe water, sanitation, and hygiene is a fundamental requirement for health and well‐being. Goal 6 of the Universal Agenda for Sustainable Development seeks to achieve equitable access to safe and affordable drinking water for all [1]. Groundwater quality is determined by physical, chemical, and biological parameters, which are influenced by both natural and anthropogenic factors [2].

Adequate consumption of high‐quality water is associated with systemic health benefits, including optimal oral health, gastrointestinal and renal function, enhanced cognitive and physical performance, and the systemic prevention of obesity [3]. The prevalence of dental caries in Mangalore, which is a part of South Karnataka, remains high, ranging from 68.1% to 82.6% [4, 5]. Groundwater in this region is often highly acidic and turbid. Sulfate and iron concentrations in this area have been recorded at levels nearly 10‐fold higher than the maximum permissible limits established by the World Health Organization (WHO) standards [6, 7].

The solubility and structural stability of dental enamel are governed primarily by the chemical saturation of oral fluids in the immediate environment. These surroundings facilitate a dynamic equilibrium on the enamel surface, characterized by a continuous cycle of demineralization and remineralization in response to localized biochemical shifts [8]. The elements are deposited in the enamel and dentin during maturation of the teeth or can be deposited on the enamel surface by a topical effect. A few trace elements, such as iron, zinc, strontium, nickel, and chromium, increase the crystallographic parameters of enamel, whereas others decrease the crystallographic parameters of enamel. This affects the hardness of enamel and contributes to caries resistance [9]. This study attempts to estimate the presence of various elements in groundwater and their correlation with dental caries. The objectives of this study are to estimate the mineral content of groundwater drinking sources in Mangalore and to estimate dental caries experience among the study population.

## 2. Methodology

A 2‐year cross‐sectional ecological study was conducted in Mangalore Taluk of Dakshina Kannada district in 2024. Ethical clearance was obtained from the Institutional Ethical Committee (ETHICS/ABSMIDS/497/2024). The study was coordinated through the institution’s oral health care delivery network, utilizing its rural satellite centers and outreach programs. In the study area, groundwater is used as a major source of drinking water in addition to public water supplies.

A multistage sampling strategy was employed to assess water quality across the study area. A sample size of *n* = 31 gram panchayats was estimated considering a 5% level of significance, power 80% and a correlation coefficient of 0.5 using nMaster 2.0 software. Mangalore Taluk has 76 gram panchayats, out of which 31 units were selected by simple random sampling using computer‐generated random numbers, as shown in Figure 1. The selected areas ensure adequate representation (40.8% coverage) of coastal, interior, and peri‐urban sectors that provide a statistically viable representation of the diverse regional geography.

**Figure 1:**
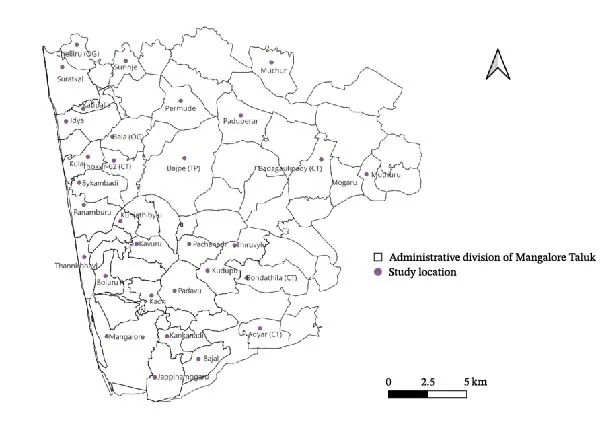
Administrative division of Mangalore Taluk.

In the second stage, a purposive sampling technique was used to identify the water source for each chosen cluster. A single water sample was collected from each of the selected gram panchayats. Since this study is a preliminary investigation to identify the relationship between minerals in drinking water and dental caries, a single representative well per selected cluster was considered feasible for establishing a baseline mineral profile of the study area. A total of 31 well water samples were collected, providing a cross‐sectional profile of the groundwater mineral content across the diverse administrative sectors of the taluk.

Adult participants aged 18–65 years were recruited from patients who were receiving community‐based oral health outreach programs within the selected gram panchayats. The inclusion criteria were limited to permanent residents who are residing in the study area for at least 5 years, provided written informed consent, and who predominantly used well water as their primary source of drinking water. To minimize exposure confounding, migrant populations and individuals utilizing multiple or mixed sources of drinking water were excluded. A total of 827 adult patients were examined from the selected 31 clusters. The socioeconomic status of the participants was assessed using B.G. Prasad’s classification system based on per capita monthly income [10].

Clinical oral examinations were performed by two examiners who were trained and calibrated by a senior public health specialist. Intra‐examiner reliability was calculated to be 0.92 and 0.94 for the two examiners, and both examiners had an inter‐examiner reliability of 0.86. Visual examination was carried out under natural light after isolating and drying the teeth with sterile cotton gauze. Examination for dental caries was performed in accordance with WHO modification of the decayed, missing, and filled teeth (DMFT) Index 1987, where a tooth is considered carious when there is an unmistakable cavity, undermined enamel, or softening of the walls or base of the cavity [11].

The water samples were collected in 500 mL of high‐density polyethylene bottles. To ensure trace metal integrity, the samples were preconditioned by soaking in dilute nitric acid for 48 h and rinsed with deionized water. A total of 13 elements were analyzed in drinking water samples. The concentrations of calcium, magnesium, barium, iron, nickel, potassium, lead, silver, gallium, copper, chromium, and cadmium were estimated by comprehensive chemical profiling, and inductively coupled plasma mass spectroscopy (ICP‐MS) was used for quantitative elemental analysis. For each of these elements, calibration curves were obtained using standard solutions, and linearity more than 0.99 was observed for almost all elements. Fluoride in the water samples was quantified using the SPADNS colorimetric method [12]. All samples were analyzed in duplicates and blanks were repeated after every 10 samples. Standard protocols ensuring quality assurance and quality controls were followed throughout the analytical procedures. The limit of detection (LOD) of each of these elements is shown in Table 1. Calibration curves of the analyzed minerals are included as a File S1.

**Table 1:** Distribution of participants by age, gender, and socioeconomic status.

Variable	Group	*N* (%)
Age group	18–35 years	245 (29.6%)
	36–50 years	267 (32.4%)
	51–65 years	315 (38%)
Gender	Female	481 (58.2%)
	Male	346 (41.8%)
Socioeconomic status (SES)	Upper class	23 (2.8%)
	Upper middle class	124 (15%)
	Middle class	452 (54.7%)
	Lower middle class	188 (22.7%)
	Lower class	40 (4.8%)

### 2.1. Statistical Analysis

IBM SPSS Version 23.0 was used for data processing. Descriptive statistics were presented as percentages for categorical variables, median, and interquartile range were used for continuous variables. The Shapiro‒Wilk test was used to assess the data distribution. Given the nonnormal distribution and high frequency of nondetectable concentrations in the trace mineral dataset, Spearman’s rank correlation was employed to evaluate the association between the gram panchayat level median DMFT and water mineral concentrations. A *p* value < 0.05 was established as the threshold for statistical significance. Given the exploratory nature of the study, we did not do a Bonferroni correction for the multiple hypotheses tested. A linear mixed model analysis was done with individuals nested within gram panchayats, with mineral concentrations assigned at the cluster level and adjustment for age, sex, socioeconomic status, and mineral covariates. Sensitivity analysis was done after removing the minerals with a detection rate less than 20%.

## 3. Results

The distribution of participants according to age, sex, and socioeconomic status is shown in Table 1. A total of 827 participants were examined for dental caries. Approximately 38% of the participants were aged 51–65 years, 58.2% were females, and 54.6% were from the middle class.

The concentrations of minerals sampled from water samples from 31 locations within the Mangalore Taluk are summarized in Table 2.

**Table 2:** Summary of mineral concentrations.

Mineral	Limit of detection	Limit of quantification	Median (Q3,Q1)	Detection (%)
Magnesium	0.7047 ppb	2.3255 ppb	0.489 (0.489,244.972)	48.38
Potassium	5.0820 ppb	16.7706 ppb	3.52 (3.52,54.808)	38.71
Calcium	1.2850 ppb	4.2405 ppb	40.56 (0.880,216.114)	64.52
Chromium	0.0248	0.0818 ppb	0.017 (0.017,0.017)	6.45
Iron	0.2145	0.7079 ppb	0.148 (0.148,0.148)	22.58
Nickel	0.0316 ppb	0.1043 ppb	0.021 (0.021,0.021)	16.13
Copper	0.0513 ppb	0.1693 ppb	0.035 (0.035,0.189)	29.03
Gallium	0.0039 ppb	0.0127 ppb	0.0027 (0.027,0.027)	3.23
Silver	0.0133 ppb	0.0439 ppb	0.0092 (0.0092,0.552)	32.26
Cadmium	0.0527 ppb	0.1739 ppb	0.036 (0.036,0.042)	32.26
Barium	0.0304 ppb	0.1003 ppb	0.0211 (0.0211,1.4905)	45.16
Lead	0.0209 ppb	0.0688 ppb	1.40 (1.40,27.80)	54.84
Fluoride	0.04 ppm	0.1 ppm	0.120 (0.070,0.140)	100

The median DMFT values for 31 locations were correlated with the corresponding mineral contents at each site in Table 3. Wherever minerals were below the LOD, they were substituted as LOD/√2 for analysis. Since the data showed a skewed distribution, the median and interquartile range were recorded.

**Table 3:** Correlation of minerals in drinking water with dental caries experience.

Mineral	*r*	*p*	95% confidence interval	
			Lower limit	Upper limit
Magnesium	−0.105	0.574	−0.509	0.279
Potassium	−0.458	0.010	−0.721	−0.123
Calcium	−0.188	0.312	−0.483	0.145
Chromium	0.271	0.140	−0.122	0.589
Iron	−0.144	0.441	−0.509	0.279
Nickel	−0.219	0.237	−0.551	0.183
Copper	−0.326	0.074	−0.701	0.120
Gallium	−0.308	0.092	−0.611	0.074
Silver	0.227	0.220	−0.126	0.543
Cadmium	−0.335	0.065	−0.616	0.033
Barium	−0.269	0.144	−0.605	0.108
Lead	−0.132	0.478	−0.467	0.205
Fluoride	0.318	0.08	−0.041	0.643

Spearman’s rank correlation analysis revealed varying degrees of association between the 13 analyzed drinking water minerals and dental caries experience (DMFT) across the 31 sampling sites. Due to high nondetection rates, minerals below the LOD were imputed as LOD/√2. While potassium showed a statistically significant negative correlation with DMFT, statistically insignificant negative correlations were observed for all elements except silver and fluoride with a DMFT score. Since dental caries is a multifactorial disease, we did a linear mixed model analysis to quantify the effect of mineral concentration, socioeconomic status, age, and gender on DMFT. Age did not have a significant association with dental caries, and participants belonging to the upper middle class had a significantly higher DMFT compared to the lower class. A sensitivity analysis was done after removing chromium, nickel, and gallium, which were detected in less than 20% sites. Among minerals, while potassium showed a significantly negative association with DMFT, none of the other minerals had a significant effect (Table 4).

**Table 4:** Linear mixed model estimates for the association between mineral concentration and dental caries experience.

Parameter	Primary model estimate	Primary model sig.	Sensitivity model estimate	Sensitivity model sig.
[Age = 61−65 years]	0.358255 (0.092707, 0.623803)	0.008	0.197321 (−0.085031, 0.479673)	0.171
[Age = 18–35 years]	−0.034358 (−0.315261, 0.246545)	0.810	−0.189367 (−0.489075, 0.110340)	0.215
[Age = 36–50 years]	0^a^	.	—	—
Magnesium	5.246047E−6 (−0.002511, 0.002521)	0.997	0.000479 (−0.000160, 0.001118)	0.142
Potassium	−0.001045 (−0.004793, 0.002703)	0.564	−0.002065 (−0.003200,−0.000930)	0.000
Calcium	0.000178 (−0.004422, 0.004779)	0.936	0.000319 (−0.000644, 0.001118)	0.515
Chromium	3.236139 (−8.190799, 14.663076)	0.558	—	—
Iron	0.004736 (−0.187598, 0.197069)	0.959	0.002573 (−0.011205, 0.016352)	0.714
Nickel	−0.848720 (−3.642249, 1.944809)	0.530	—	—
Copper	0.061832 (−0.217967, 0.341630)	0.647	0.078406 (−0.009442, 0.166253)	0.080
Gallium	−6.238068 (−15.342123, 2.865987)	0.166	—	—
Silver	0.003056 (−0.008758, 0.014870)	0.592	0.003008 (−0.001130, 0.007145)	0.154
Cadmium	−0.285744 (−9.721649, 9.150161)	0.950	−1.559912 (−4.348492, 1.228667)	0.273
Barium	0.036144 (‐−0.460919, 0.533207)	0.880	−0.026178 (−0.142470, 0.090114)	0.659
Lead	−0.003526 (−0.026354, 0.019303)	0.748	−0.000793 (−0.008369, 0.006783)	0.837
Fluoride	0.083280 (−4.735007, 4.901566)	0.971	−0.212646 (−1.891878, 1.466587)	0.804
[Gender = male]	−0.067670 (‐0.290762, 0.155422)	0.552	−0.091083 (−0.329885, 0.147720)	0.454
[Gender = female]	0^a^	.	—	—
[SES = upper class]	−0.088803 (−0.912469, 0.734864)	0.832	−0.299275 (−1.181776, 0.583225)	0.506
[SES = upper middle class]	0.846193 (0.276307, 1.416080)	0.004	0.897401 (0.285246, 1.509557)	0.004
[SES = middle class]	0.359830 (−0.157325, 0.876985)	0.172	0.419721 (−0.135589, 0.975031)	0.138
[SES = lower middle class	0.01546 1 (−0.529767, 0.560690)	0.956	0.013598 (−0.572146, 0.599341)	0.964
[SES = lower class]	0^a^	0.0	—	—

^a^Reference category.

## 4. Discussion

### 4.1. Principal Findings

This cross‐sectional ecological study provided an elaborate mineral profile of drinking water in Mangalore Taluk and correlated it with dental caries. A major finding of the study was the significant negative association of potassium ions in groundwater samples with the dental caries experience. Although not statistically significant, our study revealed negative correlations of DMFT with most of the minerals, suggesting an anticariogenic effect. The mixed model analysis revealed the same association of potassium with dental caries.

### 4.2. Comparison With Other Studies

The prevalence of dental caries observed in our study agreed with the findings of previous studies conducted in the same region. Lalithambigai et al. [4] reported a caries prevalence of 68.1% among college students in Mangalore, with a mean DMFT of 1.94. The mean DMFT in our study was 4.54, which indicates a high caries burden in the region. This high prevalence of dental caries in the region can be associated with the chemical nature of groundwater, where the fluoride content is estimated to be low. Kushalan et al. [7] analyzed open‐well water across 60 wards of the Mangalore City Corporation and reported that fluoride concentrations were consistently below the WHO permissible limit of 1.5 ppm. The suboptimal level of fluoride in drinking water necessitates a greater reliance on other protective trace minerals, such as potassium, to slow the progression of dental caries.

A negative correlation between potassium ions and dental caries was reported previously among school children by Benghasheer et al. [13]. Biologically, potassium is an essential component of the salivary buffering system, which contributes to maintaining oral pH. Potassium plays an important role in acid–base balance, which affects dietary‐related physiological functions, osteoporosis, aging, and hormonal action, and is involved in the regulation of the pH of intracellular organs, which plays an important role in the enzymatic regulation of metabolism in the human body [14]. This “whole‐mineral” approach to water chemistry is supported by the work of Razvan et al. [15], who demonstrated significant correlations between the calcium, magnesium, and fluoride contents in drinking water and the corresponding mineral values in the dentin of permanent carious teeth.

Kushalan et al. [7] identified areas with iron concentrations exceeding permissible limits in Mangalore city. Thus, the protective effects of minerals in drinking water can vary based on the geological profile of the region. Copper is recognized for its potent antimicrobial properties, which can inhibit the metabolic activity of cariogenic bacteria such as *Streptococcus mutans* [16]. Previous studies have investigated the role of cadmium and gallium in the prevention of dental caries. A study by Chen et al. [17] revealed no association between environmental cadmium exposure and dental caries. Kushalan et al. [7] reported that water samples from 20 wards in Mangalore were acidic. This can explain the lack of a protective effect of calcium in the drinking water on dental caries in our study area. Low pH can increase the solubility of enamel and override the remineralization benefits of major minerals such as calcium [4].

### 4.3. Biological Plausibility

Our study area has very low fluoride concentrations, which are far below the WHO recommended range of 0.7–1 ppm. At these extremely low levels, the protective effect of fluoride is outweighed by other factors, such as diet and oral hygiene. The mild positive correlation of fluoride, along with a negative correlation of potassium ions with dental caries, suggests that the interplay of minerals in water is more influential for the protective association against dental caries than the individual role of ions. The results of this study can be generalized to coastal developing areas such as the Mangalore taluk. However, in areas with centralized municipal water supplies, the results should be extrapolated with caution.

### 4.4. Study Limitations and Future Directions

Though a statistically significant negative association of potassium was observed, it should be interpreted with caution, and it should be considered a preliminary hypothesis‐generating study. A major limitation of this study is the ecological fallacy wherein an aggregated gram panchayat‐level dental caries experience is associated with a single well water in the study area. The significant negative relation of potassium with dental caries at the gram panchayat level may not necessarily hold true at the individual level. Since this was an exploratory study, we collected only one water sample from each representative gram panchayat. Thus, there may be variations in mineral levels caused by different soil types or well depths, and one sample per gram panchayat may not be representative. Taking multiple samples from each area in future studies would provide a more accurate correlation between water minerals and DMFT scores. Given that dental caries is a multifactorial disease, the lack of individual data on variables such as oral hygiene practices and dietary sugar intake is a limitation. Despite these constraints, the accuracy of the ICP‐MS method enabled us to identify a statistically significant correlation with potassium. These findings offer preliminary support for the potential link between dental caries and the environmental factors we measured across the entire region.

## 5. Conclusion

By integrating our findings with those of previous studies, it can be suggested that the low‐fluoride, acidic nature of the water supply in Mangalore implies the regulation of other minerals, such as potassium. Our results provide a baseline for researchers to investigate the cariogenic role of various minerals in drinking water. Longitudinal studies evaluating oral hygiene practices and diet, along with the mineral content of drinking water, are recommended to consider public health interventions to decrease the prevalence of dental caries.

### 5.1. Public Health Significance

The quality of water depends on natural and anthropogenic influences. Rapid population growth and intensive domestic activities, industry expansion, and agricultural production release many hazardous metals into the water bodies, which may adversely affect oral health. Various minerals are found to be cariostatic, but their concentrations in groundwater samples are estimated to be negligible to low. In the future, controlled trials should be directed to compare the safety and cost effectiveness of various ions in drinking water in the prevention of dental caries. This study points out the possibility that optimizing the mineral content of water supplies may serve as a contributing factor in reducing the long‐term economic burden of dental disease across diverse communities.

## Author Contributions

**Thara Chandran**: conceptualization, data curation, formal data analysis, writing original draft, project administration. **Audrey D’Cruz:** conceptualization, validation, methodology, project administration, supervision, writing – review and editing. **Radhakrishnan Kinattinkara:** conceptualization, methodology, supervision. **Mackwin D’Mello**: conceptualization, methodology, supervision, validation. **Pushparaja Shetty**: conceptualization, methodology, supervision, validation. **Neevan D’Souza:** conceptualization, methodology, supervision, validation. **Vishnu Unnikrishnan:** investigation, writing – review and editing, resources. **Herman Sunil Dsouza**: investigation, writing – review and editing, resources.

## Funding

This study was supported by Nitte University (Grant N/RG/NUFR2/ABSMIDS/2023/05).

## Disclosure

All the authors critically reviewed and approved the final draft. All content generated or refined with the assistance of ChatGPT, Grammarly, and Rubriq was critically reviewed, verified, and approved by the authors, who take full responsibility for the integrity, accuracy, and originality of the manuscript.

## Conflicts of Interest

The authors declare no conflicts of interest.

## Supporting Information

Additional supporting information can be found online in the Supporting Information section.

## Supporting information


**Supporting Information** File S1: The analytical calibration curves of all the minerals analyzed in ICP‐MS

## Data Availability

The data that support the findings of this study are available from the corresponding author upon reasonable request.
