# Supplementary material for: Correlation Between the Mineral Contents of Drinking Water and Dental Caries in Mangalore: A Cross‐Sectional Study
Source: Int J Dent. 2026 Sep 28;2026:6407047. doi: 10.1155/ijod/6407047 (PMC13618560; doi:10.1155/ijod/6407047)
Supplement: Supplementary file 1 [file IJOD-2026-6407047-s001.docx]

**Calibration graphs for minerals analysed in ICP-MS**
